# The Odontological Society of Great Britain

**Published:** 1890-06

**Authors:** 


AMERICAN JOURNAL

—OF—

DENTAL SCIENCE.

VOL. XXIV. BALTIMORE, JUNE, 1890. No. 2.

ARTICLE I.

### THE ODONTOLOGICAL SOCIETY OF GREAT BRITAIN.

The usual monthly meeting of the above Society was held on the 5th ult., at 40, Leicester Square.

The President, Mr. Felix Weiss, L. D. S. Eng., occupied the chair, and there was a very full attendance of members.

The minutes having been read and confirmed, Messrs. J. O. Butcher, Jas. F. Colyer, Jno. Greenfield, Chas. F. Rylot, and R. Wynn Rouw were balloted for, and duly elected members of the Society.

The Librarian (Mr. Ashley Gibbings) announced the receipt of various books and publications; among them being an Italian work, the English title of which was "The Pathology, Therapeutics, and Hygiene of the Cavities of the Mouth," the Year Book of the Scientific Societies, and a new Journal from Turin.

The Curator (Mr. Storer Bennett) had no report to make.

Mr. T. G. Read made a communication on "A method of making an all-gold crown, illustrating a convenient way of making and using a model." He said that a model was most useful when crowning bicuspids and molars, and in some cases incisors and canines. The metal band of the crown is roughly adapted to the stump, and feather-edged previous to fitting in the mouth, the portion passing under the gum is the same relative distance beneath it around the stump, and a very perfect occluding surface is obtained. When crowning a stump, the rubber dam should, if possible, be first adjusted, and the pulp canals filled. The broken-down crown should then be reduced in height to allow for restoration of the occluding surface, the stump being left standing as high as possible above the gum Should it be necessary to cut away much tooth tissue, a long file cut fissure bur with a chisel point would be found useful for that purpose; drill two holes with the point for the labial to the lingual surface, one at the mesial, the other at the distal part of the crown. The tooth substance between these holes having been cut away, one blade of a pair of excising forceps is placed in the labial, and the other in the lingual opening, and the handles pressed together when the crown comes away. Small pieces of upstanding tooth substance close against another tooth may be readily removed with a wheel bur such as Dr. Meriam's, these projections can be cut from the inside without wounding the gum, and the unpleasantness of running a corundum wheel is avoided. The sides of the stump as far as the band is to extend should be made quite parallel, so that the crown may fit the stump quite closely and tightly. Previous to to paring the stump, cocaine in crystals should be rubbed on the gums with the finger, then the enamel and overhanging or projecting tissue may be stripped off with suitable enamel chisels. The sides of the stump having been pared quite parallel, should be finished smooth by carefully passing a safety point shouldered fine file cut fissure bur around it. Then take a strip of thin metal—such as telephone plate—trim and bend this to the stump, when roughly fitted, press a small piece of softened composition to the band of stump, the patient then closes the mouth, and bites into the composition. As soon as it is hard, remove the impression of little band from the mouth. Cast a lower and upper model from this with the little band *in situ*, over this band make the metal band of the crown in coin gold, size 5, to fit it and correspond to the gum edge; the join should be at the lingual surface. Having fitted the band to the model, soften the end of a stick of composition and press the band on with the edge to go under the gum uppermost, this is feather-edged with a fine round file. Coin gold of the same substance of the band should be used for the cusps which should be struck up in White's die plate. Try the cusps to the occluding model and see if the bite will ride. Mark where it will, place the cusps on the male die of soft metal used to strike them up and with blunt punches knock down those places marked. The articulating surface is thus made perfect. Fill up some solder and mix it with a little Parr's flux, fill the interior of the cusps rather full and flow the solder over a Bunsen flame. The band should then be fine, fitted in the patient's mouth; when this has been done, solder it edge to edge over a Bunsen flame using binding wire as a clamp. Contour the band with contouring pliers. If the canals have not been filled, twist a piece of binding wire with a bead or two upon it round the contoured band, place this on the stump and use it to hold the rubber dam. Soften the end of a stick of composition and press the band upon it, with the occluding edge uppermost, with a fine flat file. Cut the surface flat, remove the band from the composition, and try it and the cusps in the mouth, removing and replacing it upon the stick to cut away until the cusps are let in and the occlusion is perfect. Place the cusps on a soldering gridiron, borax the edge of the band, and adjust it in position on the cusps, so that when the shoulder which is on the cusps is cut away, the buccal and anterior surfaces will be perfect. Hold the work over a bunsen flame, and the solder in the cusps will melt and unite with the band. When soldered, if the lingual and posterior surfaces are not perfect, build them up with coin gold, scraps, and solder filings; run the solder with a blow-pipe. Boil in acid, trim with a fine corundum wheel, and polish, holding the crown on a stick of composition. Horizontally groove the pulp chamber, dry it out and fill it and the anterior of the crown with oxy-phosphates of a creamy consistency, press the crown on the stump with a notched tooth-brush handle, strike the tooth-brush handle once or twice with a lead mallet to expel any surplus cement. When the setting is hard, trim away any excess of cement with a broken-back chisel. The mallet should be only used in the final stage, for, if properly made, the band may be pressed on the stump with the finger.

Mr. Walter H. Coffin remarked that in hearing a communication read with the rapidity that the one they had just listened to had been given to the meeting, it was quite possible to overlook some points. The only new point which he had been able to see was the fitting a base metal immediately, and adapting around that the permanent gold crown which would of course be too large, and then have to be closed up in the mouth and soldered. It seemed to Mr. Coffin that the filling of the permanent gold crown on the stump could be accomplished in the first instance as easily as in any other metal; he imagined, however, that Mr. Read found it more convenient to handle some other metal in the initial stage.

Mr. Read said the telephone plate band was not fitted to a nicety but only roughly; it could be done in about three minutes. Then the band was cast, or an impression taken, and an assistant could get the gum edge right. One advantage was that an assistant could do the work which would otherwise have to be done by the principal himself. Then the point was to get the gum edge right, in the specimen he had passed round, it was very shallow on one edge and deep on the other, it would be very difficult to make it in any other way.

Mr. H. Baldwin narrated a case of chronic enlargement of the upper jawbone which had been under his observation for about eight years. The patient was an unmarried woman aged 39, and on first coming to him the enlargement was very marked, chiefly on the left under side of the molar region. For about six years after he first saw her it underwent very little change, but in the seventh year the enlargement grew considerably and the mucous membrane became pencilled over with blue veins, suggesting that the circulation was getting more free, indicating the necessity for operation. The growth was hard and bony to the touch. He had called it a case of hyperostosis, for want of a better definition, as it had many of the characteristics of that disease. The case was operated upon in the latter part of last year by Mr. Pearce Gould of Middlesex Hospital, the gum was deflected away from the growth, and the bone removed with bone-cutting forceps. It was found very easy to remove, and not of the ivory description which he had anticipated. The peculiarity of the case was, that wherever the partial plate, which the patient was wearing, touched the mouth, there the growth was much less marked; for several years everywhere where the plate touched was perfectly healthy, it was only in places where there was no contact with the plate that the growth developed. The plate seemed to have operated in preventing the occurrence of the growth and in no way to have irritated it. The model, which he showed, had been taken since the operation, and as that was performed only in December last, sufficient time had not elapsed to say whether a complete cure had been effected, but at present there were no signs of recurrence of the growth. One peculiarity of the patient was that the nasal processes of the maxillary bones were more prominent than is normal, and the question was whether this was a first instalment of a general enlargement or whether the nasal prominence were merely accidental, which would be cured by operation. There were sections of the growth under the microscope; it seemed to consist of a cancellous bony structure and the spaces were lined with fibrous membrane. Portions of the growth contained small cysts visible to the naked eye, about the size of a very small pea, lined with glistening membrane.

Mr. Howard Mummery then read a paper entitled—

### NOTES ON THE PREPARATION OF MICROSCOPICAL SECTIONS OF TEETH AND BONE.

The paper was accompanied by an exhibit of photo-micrographs thrown upon a screen by a lantern. Having referred in some detail to the disadvantages of the "decalcification method," Mr. Mummery said that his interest in the process he was about to describe was first awakened by a remark of Mr. C. S. Tomes, who told him some two years ago that Professor Moseley, of Oxford, had mentioned a plan of hardening sections of teeth with gradually increasing strengths of alcohol. No details, however, of the process were given by him. But these particulars were supplied by Dr. L. A. Weil, in a German Microscopical Journal, and were reproduced as an extract in the English Royal Microscopical Society's Journal, December, 1888, p. 1042.

Mr. Mummery prepared some sections according to those directions, and was so pleased with the results that he had since cut nearly 200 specimens in the same way. By this method no decalcification is required, and the cells and connective tissue of the pulp, and also of the peridental membrane, are retained in their natural relations to the hard tissue.

The extract in the Microscopical Society's Journal stated that:—"Dr. L. A. Weil takes only fresh or nearly fresh teeth, and in order to allow reagents and stains to penetrate into the pulp cavity, divides the tooth immediately after extraction with a sharp fret saw below the neck into two or three pieces, allowing water to trickle over it the while." Mr. Mummery said that to procure longitudinal sections it was advisable to cut them a little to one side of the pulp cavity, just opening this enough to enable stains to penetrate. Continuing the quotation, "The pieces are then laid in concentrated sublimate solution to fix the soft parts. The sections are then washed in running water for about an hour and placed in 30 per cent. spirit for twelve hours, and for a corresponding period in 50 per cent. and in 70 per cent. spirit. To remove the black sublimate precipitate the teeth are then laid for twelve hours in 90 per cent. spirit, to which 1.5 to 2.0 per cent. of tincture of iodine has been added. The iodine is removed by immersion in absolute alcohol until the teeth become white. They are now ready for staining, and the stain which Dr. Weil recommends is borax carmine (alcoholic or aqueous solutions). After being washed for fifteen to thirty minutes in running water, they are left in the stain for two or three days; they are then transferred to acidulated 70 per cent. spirit, in which they remain, the watery-stained ones at least twelve, the alcoholic-stained ones twenty-four to thirty-six hours. They are then immersed for 15 minutes in 90 per cent. spirit, and then for half an hour in absolute alcohol, after which they are transferred to some etherial oil for twelve hours or more. The etherial oil is quickly washed off with pure zylot, and they are placed for twenty-four hours in pure chloroform; after this they are passed into a solution of balsam in chloroform. This balsam is prepared by drying in a water bath, heated gradually up to 90 degrees C. for 8 hours or more, until when cold the balsam will crack like glass on being punctured. The sections are allowed to lie for twenty-four hours in a thin solution of this dried balsam in chloroform, and then as much balsam is added as the chloroform will take up. The sections covered with the balsam solution are then placed in a suitable receptacle over a water bath kept at 90 degrees C. and this cooking is kept up until the mass of balsam, with the teeth in, cracks like glass when cold. This requires two or three days. Thin pieces are then cut from them with a sharp fret saw, and they are then ground down in the usual manner"—first on a corundum wheel, afterwards on stone. Mr. Mummery said that the advantage of the sublimate appeared to be due to its coagulating the albumen of the tissues—it certainly seemed to be very efficacious in preventing shrinkage. His most successful sections had been ground down on a Washita stone, using a piece of cork, or the finger, and plenty of water. The *debris* could be very conveniently washed off the completed sections with a fine spray of water, blown through an ether spray apparatus. The section could then be mounted in chloroform balsam.

The process as detailed no doubt appeared very tedious and complicated, and was almost enough to deter anyone, who had but little leisure, from undertaking it; but when a number of sections were being prepared in different stages the passing on from one solution to another did not occupy much time. Wolrab's gold bottles in a rack formed excellent receptions for the sections, a note being made on a label on the bottle of the stage they had reached. With this as with most other processes, there were of course a good many failures, some being caused by insufficient cooking, resulting in the pulp not being hardened enough, others by too prolonged cooking, which produced brittleness. The cutting down was certainly very tedious, and should be done on a slow-cutting stone. Again, without great care in grinding very thin sections, the pulp might break away at the last moment—only by practice could one learn to avoid this annoying accident. Borax carmine penetrated well and stained the nuclei very strongly, but did not give so much detail in the pulp as some other stains. Very good results might be obtained with aniline blue-black, which stained the nerve fibres as well as the nuclei and connective tissue. Mr. Mummery had not been very successful with hæmatoxylin, but had been told that Erlich's hæmatoxylin, which does not precipitate, would probably be the best stain to use in this process. The teeth he had made use of had been chiefly young bicuspids, some with the apex of the root still incomplete. He had also made sections of older teeth for comparison, and of some carious teeth and abscesses. In these latter he thought the process might prove very useful, enabling one to study the early stages of abscess formation.

Mr. Mummery, having expressed his indebtedness to Mr. Theodore Harris for the great help he had given him in preparing the sections, devoted the remainder of the time to pointing out the characteristics of the very beautiful specimens photographed on the screen. No description of these specimens will convey any adequate idea of their appearance, but a reference to one or two will serve to demonstrate the importance of the process.

A transverse section of the pulp of bicuspid tooth exhibited the pulp with its relation to the walls of the pulp cavity undisturbed. The odontoblast layer was seen very distinctly differentiated from the rest of the pulp, lying in immediate contact with the semi-calcified portion of the dentine, the tissue, "on the border-land of calcification," that part of the matrix which had evidently undergone some change, being in advance of the line of complete calcification. The blood vessels were seen in transverse section, and also the slightly denser condition of the central part of the pulp, noticeable in many of the specimens.

The next slide from a similar pulp was interesting as showing in the large blood vessels in the centre what very delicate tissue could be retained in position by the hardened balsam during the process of grinding.

Another slide, taken from a tooth which was extracted before the apex of the root was completed, showed the odontoblast cells, which, together with their nuclei, had taken the stain deeply, not to be lying in close contact but to have distinct spaces between them. Mr. Mummery did not think this was due to shrinkage in preparation, as he had found it in all the open ended bicuspid teeth which he had examined; and other specimens prepared by this process seemed to indicate that there is no appreciable shrinkage of the odontoblast cell. Mr. Hopewell Smith, in a paper published in the *Dental Record* for August, 1889, speaking of the dentine after the commencement of calcification said: "Between some of the cells of the membrana eboris there are wide visible spaces, filled with homogeneous substance and small round and angular cells." Mr. Mummery remarked that Mr. Tomes also appeared to be a little shaken in his views on this point, for whereas in the earlier editions of his "Dental Anatomy," he said (p. 159, second edition): "The odontoblasts are fitted closely together, and there is no room for any other tissue between them"; in the third edition (p. 69) he says: "There is not much room."

Deposits of secondary dentine in the pulp are well exhibited by this method of preparation. The specimen shown was taken from a molar tooth, to all appearance sound, which caused intense neuralgia, rendering it necessary to extract it. The pulp was densely packed with secondary deposits, encroaching in every direction upon the nerves and blood-vessels. This deposit exhibits some curious concentric and radiating masses. The next slide was from a similar pulp, showing some very large deposits.

Another showed a tooth extracted from an old person, in which the whole of the pulp appears to be converted into a semi-calcified material, apparently of cartilaginous consistency, with islands of calcified tubular dentine. Mr. Mummery had been struck with the fact pointed out by Mr. Salter in his "Dental Pathology"—that many young and apparently healthy pulps show numerous deposits of secondary dentine. Mr. Salter, in the work referred to (p. 139), says this change is to a great extent reparative, and the result of trivial causes, though Mr. Mummery believes it never occurs unless the tooth has been in some way the subject of injury or irritation.

The specimens in which it was seen were certainly untouched by caries; but they may have been subjected to some form of irritation conveyed to the pulp from the great pressure caused by overcrowding.

Interglobular spaces in dentine are very well stained in the balsam process.

Caries in a fissure in the enamel.—This slide was prepared to show that the process, while keeping the relations of the carious portion to the calcified tissue, retains also in position tissue that has undergone a very considerable amount of disintegration.

A great number of slides were shown; and in conclusion Mr. Mummery expressed the hope that some of the points he had simply touched upon might suggest lines of investigation to those engaged in microscopical work.

The President said they were greatly indebted to Mr. Howard Mummery for his very lucid and able paper, and for the beautiful illustrations, and he invited comments upon the paper.

Mr. F. Newland-Pedley said he was able to confirm from his own experience the value of the process which Mr. Mummery had described, but like many other things, it was of some antiquity, having been known for the last fifteen years. Some five or six years ago one of his colleagues at Guy's wished to investigate the development of rider's bone, and it was necessary to show hard and soft structures at the same time: he (Mr. Newland-Pedley) cut sections by Mr. Mummery's method, and they were shown at the Pathological Society.

Mr. H. Baldwin wished to ask Mr. Mummery whether the transparent portion which intervened between the dark layers was not chiefly formed of the unstained portion of the odontoblast cell, and whether the dark layer did not consist of the nuclei only of the odontoblast. Also whether the so-called spaces were not unstained portions of the cells? Again, as to the small groups of cells which were found in the periosteum, and which were said by some to be epithelial pearls, whether there were not some stain which would show whether they were of an epithelial nature or not?

Mr. Arthur Underwood said if it would not be taking a liberty to relieve Mr. Mummery of the trouble, he would take upon himself to answer two of the questions. If the parts were stained with gold the whole cell becomes perfectly stained, and the interspaces are quite marked, and that without confusion of substances, still more the transparent layer that lies between the cells is marked out quite plainly, being in no kind of sense a part of the cell, there could be no kind of confusion, there was no doubt that an interval does exist. Mr. Underwood thought that they ought really to feel very much indebted to Mr. Mummery for his epoch-making paper in dental microscopy. Though Mr. Newland-Pedley seemed to have been so happy as to have hit upon the same process a long time ago, still it had remained for Mr. Mummery to make it public property. All dental microscopists would feel under a great debt of gratitude for having solved the difficult problem how to cut hard and soft tissues together leaving them undisturbed by the influences either of the knife or the fluids. Mr. Underwood knew from experience how ready critics were to assert that the appearances were due to decalcifying fluids. He thought a tablet should be raised to Mr. Mummery for having delivered them from these tiresome critics.

Mr. Charles S. Tomes wished, in endorsing Mr. Underwood's remarks, to emphasize the fact that Mr. Mummery had been the first to produce preparations which would go very far towards necessitating a revision of much that had been written on the question of the development of dentine. The points in question Mr. Mummery had hardly touched upon, because until he had thoroughly worked the subject out he very rightly did not wish to say anything he might have to recede from. Mr. Tomes would not have said anything about it had not Mr. Mummery confined himself very much to the exhibition of the process, and had not Mr. Newland-Pedley said that the process was not very new, thereby implying that it was not worth while demonstrating what the process could do. This much he would assure Mr. Newland-Pedley that Mr. Mummery's demonstrations were entirely novel to him (Mr. Tomes); they showed things which he had never seen approached, and he felt sure they would give some results which would necessitate a great deal of re-writing.

Mr. George Cunningham desired to call attention to the fact that the photographs were all the work of Mr. Mummery himself, so that he was not only an able microscopist but also a photomicrographist who might vie with Mr. Andrew Pringle who had been described as *facile princeps*.

Mr. Charters White felt that he ought to add his testimony in favor of Mr. Mummery's very able paper. He (Mr. White) had been reading for the last thirty years on the subject of microscopy and photomicrography, and had made the subject a special study, but he was bound to say that he had never been so fortunate as to reach the process before. Decalcification of the bony tissues resulted in the destruction of the shape of the cells, and the presence of acid in the pulp made them very difficult to stain. Mr. White felt that Mr. Mummery had given the death knell to the decalcification of the tissues, and he would, for one, adopt the process new to him, because he felt it was capable of giving details which decalcification had never yet afforded. He would like to ask Mr. Mummery if the sections could be rubbed down in his process in the same manner which he (Mr. White) had always described for dry sections, because in that way it would be possible to get photographs much clearer and sharper.

Mr. William Hern wished to ask Mr. Mummery if he could explain how it was that after a process of prolonged and powerful heating, soft tissue, which was known to contain a large per centage of water, seemed to occupy the same space as before the process.

Mr. Charters White said, if he might be allowed to reply, he thought the use of the corrosive sublimate as a fixing agent, and then afterwards the hardening in absolute alcohol prevented, any further change, for the soft tissue being saturated with Canada balsam could not possibly have its real histological elements altered.

Mr. Howard Mummery said, in reply to Mr. Newland-Pedley, that he was not aware that the process had been really well known before, although of course there had been hints of it, but he thought to Dr. Weil belonged the credit of bringing it out properly, and giving those minute details which were so necessary to practical working. With regard to Mr. Baldwin's questions, Mr. Arthur Underwood had so ably answered them that he did not think it was necessary for him to add anything. He (Mr. Mummery) quite agreed with Mr. Underwood that the spaces between the odontoblast layer and the fully calcified dentine are crossed by the tubes, and the spaces between the cells are not part of the cells. If Mr. Baldwin would look at the specimens under the microscope he would see that this was so. Mr. Mummery wished to thank Mr. Tomes for his very kind remarks. He quite agreed with Mr. White as to the evil effects of decalcification, and thought that the specimens might be cut down by his (Mr. White's) method, but at present he was only feeling his way, and had not adopted it; the difficulty would be to be able to see if the pulp were sufficiently rubbed down. With regard to Mr. Hern's remarks, the water in the tissue is gradually taken out by the spirit, the shrinkage is avoided by slightly increasing the alcohol, and the balsam takes the place of the spirit. The value of the corrosive sublimate is very great, as it fixes the specimen and prevents the shrinkage which often takes place.

The usual votes of thanks having been passed, the proceedings terminated with the announcements for next meeting.—*London Dental Record.*

